# A simple method for the synthesis of biochar nanodots using hydrothermal reactor

**DOI:** 10.1016/j.mex.2020.101022

**Published:** 2020-08-12

**Authors:** Fuyu Guo, Li Bao, Hanrui Wang, Steven L. Larson, John H. Ballard, Heather M. Knotek-Smith, Qinku Zhang, Yi Su, Xingxiang Wang, Fengxiang Han

**Affiliations:** a Department of Chemistry and Biochemistry, Jackson State University, Jackson, MS, United States; b Institute of Soil Science, Chinese Academy of Sciences, Nanjing 210008, China; c U.S. Army Engineer Research and Development Center, Vicksburg, United States; d Department of Chemistry, University of Houston, Clear Lake, Houston, TX, United States

**Keywords:** Nano-biochar, Soybean straw, Cattle manure, Hydrothermal method

## Abstract

Biochar is a stable carbon rich by-product synthesized through pyrolysis of plant and animal based biomass, and nano-biochar material has gained increasing attention due to its unique properties for environmental applications. In the present study, a simple cost-effective method for the synthesis of biochar nanoparticles through hydrothermally using agricultural residuals and by-products was developed. Both soybean straw and cattle manure were selected as the feedstock to produce the bulk-biochar. The synthesis procedure involved the digestion of the bulk-biochar with concentrated nitric acid and sulfuric acid in a high pressure condition using a hydrothermal reactor. The suspension was isolated using vacuum filtration with 0.22-μm membrane followed by drying at 65 °C in an oven. Scanning electron microscopy results revealed that both of the biochars had a well-developed porous structure following pyrolysis. Both transmission electron microscopy and the dynamic light scattering results of the hydrothermally treated biochar indicated that the soybean straw and cattle manure biochar nanodots had an average of 5-nm and 4-nm in size, respectively. Overall two raw materials produced 8.5–10% biochar nanodots. The present method presents a simple, quick and cost-effective method for synthesis of biochar nanodots. The method provided a useful tool discovering the applicability biochar nanodots for environmental applications.

• Nano-biochar formation from bulk-biochar using hydrothermal reactor

• Evaluate nano-biochar's environmental fate and behavior in soil and water

• Synthesize multifunctional adsorbent using nano-biochar as primary material

## Graphical abstract

**Figure:**
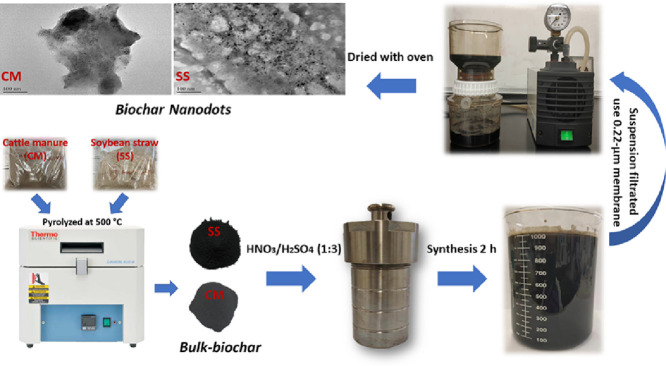

**Specifications table**

**Table:** 

Subject Area	Environmental Science
More specific subject area	*Heavy metal pollution control and remediation*
Method name	*A simple method for the synthesis of biochar nanodots using hydrothermal reactor*
Name and reference of original method	*M. Ahmad, A.U. Rajapaksha, J.E. Lim, M. Zhang, N. Bolan, D. Mohan, M. Vithanage, S.S. Lee, Y.S. Ok, Biochar as a sorbent for contaminant management in soil and water: a review, Chemosphere, 99 (2014) 19–33. [**2**]* *Y. Gao, A. Pramanik, S. Begum, C. Sweet, S. Jones, A. Alamgir, P.C. Ray, Multifunctional Biochar for Highly Efficient Capture, Identification, and Removal of Toxic Metals and Superbugs from Water Samples, ACS omega, 2 (2017) 7730–7738. [*11*]*
Resource availability	*N/A*

## Method details

### Background

Biochar is a solid material obtained from thermochemical conversion of biomass in an oxygen-limited environment [1]. Biochar's usefulness as a sorbent material in the pollution control in the water and soil ecosystem results from its porous structure. Biochar has been described as an efficient sorbent for the removal of organic and inorganic contaminants from wastewater [2]. As an amendment for soil, studies have described biochar's effectiveness for immobilizing heavy metals in soil, such as cadmium, and reducing their accumulation in plants [3,4].

The composition and structure of a material determines its characteristic and application. The pyrolysis condition and feedstock types are the two main factors controlling biochar's properties [2,5]. The carbonization temperature plays a vital role in the structure of biochar products. It has been shown that, as the carbonation temperature increases, the nano-pore size of the biochar increased, however, some microporous structures on the surface of biochar may be destroyed when the carbonation temperature exceeded 700 °C [6]. Generally, bulk-biochar (0.04–20 mm) are often directly produced and employed for pollution control in the remediation project. Recently, a number of studies have found that bulk-biochar was physically degraded into nanoscale particles after entering into the environment [7,14]. Currently, the fate and behavior of bulk-biochar in soil and water ecosystem, depending on feedstock and pyrolytic temperature, have been extensively studied. However, the size of biochar might be an important factor yet overlooked in determining its properties and environmental fate.

Carbon nanomaterials, such as carbon nanotubes, graphene, carbon dots and fullerene, have a large surface area. Functional groups on the surface or at the edge of the materials layers with a superior adsorption capacity and efficiency for various metal ions [8]. It is reported that nano-biochar with the size smaller than 100-nm had led to increased mobility in water and soil environment compared to micro sized biochar from grinding or crushing biochars [9]. As a carrier, nano-biochar could facilitate the migration of natural solutes and contaminants, in contrast with the effects of bulk-biochar in holding nutrients and immobilizing hazardous chemicals [5]. Only adsorption was observed on bulk-biochar, while not only adsorption but also fragmentation of eDNA molecules was found to occur on nano-biochar [10]. Gao et al. [11] developed the biochar dots of an average size of 3–4 nm and used that material to synthesize multifunctional fluorescence magnetic biochar that were used to remove metals and pathogens from water samples. The results showed that more than 97% of cobalt (Ⅱ) was removed from water by the multifunctional biochar and also demonstrated that it was a highly efficient adsorbent of the methicillin-resistant *staphylococcus aureus*. Therefore, development of techniques for the conversion of bulk-biochar to nano-biochar have a promising application in soil and water remediation [7]. However, to date, no universal method for the synthesis of nano-biochars was proposed.

In this study, a simple method for the synthesis of biochar nanodots using hydrothermal reactor was reported and may provide a useful tool for better understanding of the environmental behavior of nano-biochars.

### Procedures

1. Grind the bulk-biochar using a commercial blender
2. Pass the ground biochar through a 60-mesh sieve
3. Add 1 gram of homogenized, < 60-mesh sieve size biochar in a hydrothermal reactor
4. Add 15 mL concentrated HNO_3_ and 45 mL concentrated H_2_SO_4_ per gram of the ground biochar
5. Static allow reaction at room temperature for 2 h in the fume hoods
6. Transfer the mixture from the hydrothermal reactor to a beaker filled with 1000 mL deionized water, stir 1 min by glass rod and stand 10 min
7. Remove the solids part and filtrate the suspension using the vacuum filter with 0.22-μm membrane
8. Put the filter membrane (covered with biochar nanodots) to the oven dried at 65 °C for 48 h

### Final remarks

The present work describes a facile hydrothermal procedure scalable synthesis of biochar nanodots. The as-obtained biochar nanodots could be used within studies of environmental applications or as a primary material prior to further functionalization to further enhance their use as sorbent material. The mechanisms of transformation under hydroscopic acid digestion are attributed to the ratio of the concentrated acid used to perturb the microstructure of biochar in the oxidizing, high pressure and temperature aqueous environment. Compared to other methods, such as grinding which requires high energy consumption, and sonication only produces particle sizes that are larger than those of the hydrothermally produced nano-dots. Also, the method is useful for evaluating optimal conditions for nano-biochar synthesizing by systematic changes in variables such as which temperature, time, and aqueous environments used during the hydrolysis setup. Therefore, as a means of producing biochar nano-dots for studying their environmental fate and behavior in the soil and water ecosystem the described synthesis method proved to be simply, quick and cost-effective (Table 2). In the future, the effects of biochars’ properties on the synthesis of biochar nanodots need to be further investigated.

### Biochar preparation

Biochars of interest from a wide range of feedstock, pyrolysis temperatures, gas-phase components, and activation chemistries. In this description both of the feedstock for the biochars used were soybean straw and cattle manure. The two agricultural-biomass were collected from a farm in the city of Clinton, Mississippi, US. After air-dried, crushed, pulverized and sieved through a 10-mesh sieve, subsamples of both materials were then respectively pyrolyzed in a tube furnace (Thermo Scientific Lindberg/Blue M mini-mite tube furnaces). The pyrolysis temperature was set as 500 °C. The pyrolysis atmosphere was nitrogen and gas flow rate was 5 mL/min. The heating rate was set as 5 °C/min and the residence time was 2 h. After natural cooling, the bulk-biochar product was thus obtained.

### Biochar characterization

The surface morphology of both bulk-biochar and nano-biochar were characterized using the scanning electron microscopy (SEM, Tescan-Lyra3) and the transmission electron microscopy (TEM, Jeol-1011). Average size of the both nano-biochar suspensions were determined by dynamic light scattering (DLS, Malvern Nano-ZS). The pH of nano-biochar was measured at biochar: water = 1:5 (w/v) using a benchtop pH meter (Star A211, Thermo Scientific).

### Verifying the validity

**Fig. 1:**
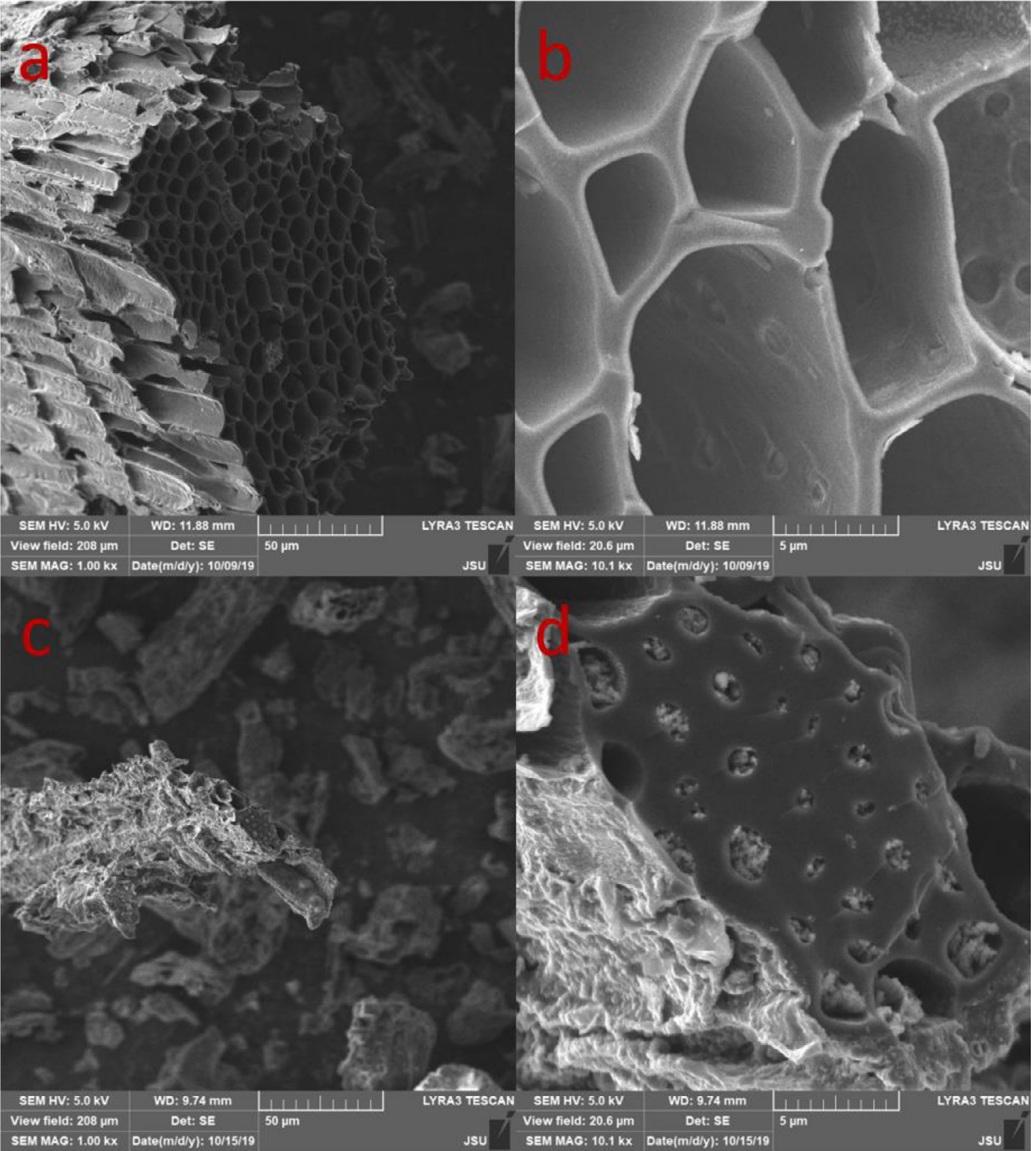
SEM images of bulk-biochar of soybean straw (a, b) and cattle manure (c, d) pyrolyzed at 500 °C.

The SEM images of both biochars were presented in Fig. 1. The results showed that the surface of both biochars was rough with a repeating pore structure. The soybean straw-biochar contain mostly empty pore channels while the pores of cattle manure-biochar often contained ash. Moreover, batch adsorption experiment results showed that the uranium adsorption capability of the soybean straw-biochar (Fig. 1a, b) was higher than that of the cattle manure-biochar (Fig. 1c, d) (data not shown).

**Fig. 2:**
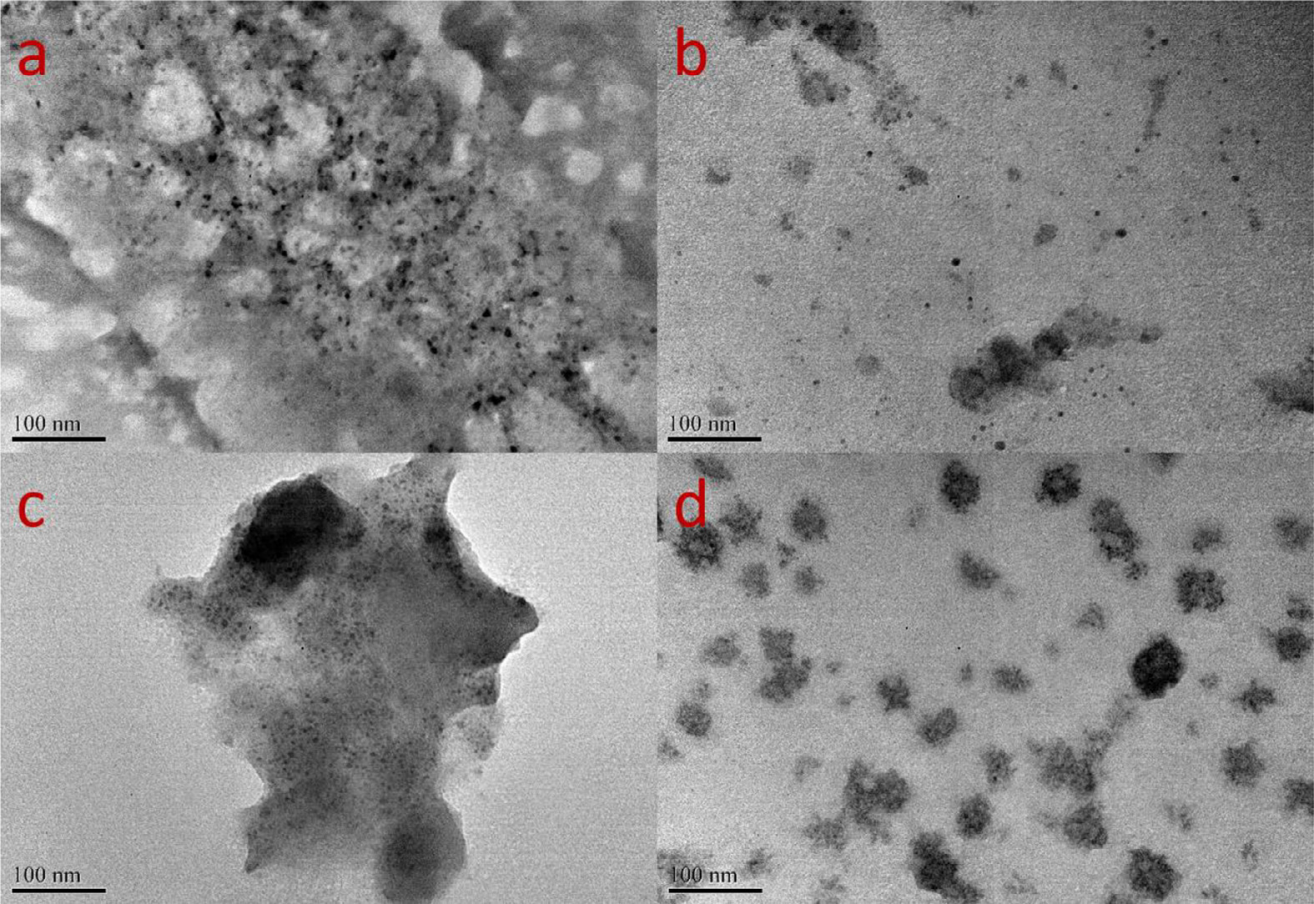
TEM images of biochar nanodots of soybean straw (a, b) and cattle manure (c, d).

The TEM images showed that biochar nanodots were produced (Fig. 2). Both TEM and DLS measurements showed that both biochar nanodots were dots of 2–10 nm in size. Carbon nanodots have attracted considerable attention as a promising alternative to semiconductor quantum dots due to their low-cost and intrinsic low toxicity. Ming et al. [12] reported a facile electrochemical approach for the preparation of carbon nanodots (3–6 nm) and the synthesized dots showed peroxidase mimetic behavior and photo catalytic. Also, Chen et al. [13] synthesized nitrogen-doped carbon dots (N—CDs) and the results indicated the N—CDs enhanced bioaccumulation efficiency and tolerance for cadmium (Ⅱ) in *Arabidopsis thaliana*.

Biochar is a stable carbon-rich material. Thus, its degradability in environment was usually overlooked. However, studies observed the physical degradation of bulk-biochars into nanoscale particles [14]. Also, the pristine pyrolyzed biochar had a wide size range and the percentages of nanoparticles ranged from 1.6% to 2.6% [9]. In comparison with bulk-biochar, nano-biochar has excellent mobility in the soil matrix and can even transport from terrestrial to aquatic environments via infiltration and surface runoff [15]. Recently, Lian et al. [10] found that nano-biochar could trigger decomposition and transformation inhibition of antibiotic resistant genes in aqueous environments while bulk-biochar played a negligible role, which highlight the importance of the size effect in evaluating the reactivity and related environmental risks of biochars. Therefore, a cost-effective method for the synthesis of biochar nanoparticles is urgent to help comprehensively understanding the biochars’ application in practical. Chen et al. [9] prepared the biochar nanoparticles by grinding method and Wang et al. [16] reported the biochar nanoparticles by sonication method. Overall, it is believed that grinding and sonication are two effective approaches to simulate physical weathering of biochar in natural conditions [7]. Herein, the hydrothermal method as previously reported for carbon dots synthesis [17] was applied to synthesized biochar nanodots and the current results showed that the yields of biochars nanodots from bulk-biochars were 10.1% (soybean straw) and 8.6% (cattle manure) (Table 1). In conclusion, the proposed method was cost-effective in reusing agricultural residue, and simple and easy to operate. The hydrothermal reactor and all reagents were commonly used in laboratory. Therefore, biochar produced using agricultural-residue/by-products was a sustainable management strategy for addressing the food, environment, and energy crisis [21].

**Table 1:** Yields, size distribution and pH of the synthesized biochar nanodots.

	Yield^a^ (%)	DLS size (nm)	TEM size (nm)	pH
Soybean straw	10.1 ± 0.5	5 ± 3	5 ± 4	8.6 ± 0.3
Cattle manure	8.6 ± 0.3	4 ± 2	4 ± 3	8.9 ± 0.4

a mean size ± standard deviation.

**Table 2:** Comparison of methods for the synthesis of biochar nanodots.

Method	Feedstock	Pyrolysis Temperature (°C)	Main Procedures	Size (nm)	Yield (%)	References
Sonication	pine wood, wood chip, barley grass, wheat straw, peanut shell, rice husk, dairy manure, pig manure, sewage sludge	500	Bulk-biochar was added at a ratio of 3% with deionized water, stirred 1 min, ultrasound 30 min, settle quiescently 24 h, suspension centrifuged at 3500 g for 30 min, supernatant freezing-dried to obtained the nano-biochar powders.	<100	0.99–15.3	[18]
Sonication	wheat straw, pine needle	350, 550	Bulk-biochar was mixed with deionized water, stirred, and sonicated 30 min to prepare biochar nanoparticle stock suspension. Nano-biochar suspension was isolated by passing the stock suspension through 100-nm pore size membranes.	98–126	1.6–2.6	[16]
Sonication	peanut shell	300, 400 500, 600	Bulk-biochar was mixed with deionized water, dispersed 15 min at 25 °C under sonication at 120 W, settle quiescently for 24 h. The nano-biochar was retained by centrifugation of the remaining suspension at 4200 g for 30 min.	17.4–25.3	0.47–2.36	[7]
Grinding	wood chip	500	Bulk-biochar was ground into powders using a ball grinder, and passing through a 200-mesh sieve.	4	Not provided	[9]
Grinding	hardwood	350	Bulk-biochar was added at a ratio of 10% with stainless steel ball, rotated at 500 rpm and 6 h in half cycle of pause time 15 min per 30 min interval.	260	Not provided	[19]
Grinding+ sonication+ centrifugation	tree branch	350, 450 550	First, bulk-biochar was placed into a planetary ball mill, which operated with a mass ratio of ball: biochar being 1: 20 at a speed of 350 rpm for 2 h, sieved using 0.25 mm sieve. Then, the powder was mixed with deionized water, sonicated with probe-type cell disruptor at 450 W for 1 h, stirred 1 h, sieved by 0.05 mm sieve. Final, the sieved suspension centrifuged 20 min at 9500 rpm, the upper supernatant was collected.	1–100	2.16–3.19	[20]
Hydrothermal	Soybean straw, cattle manure	500	See the *Procedures* part.	4–5	8.6–10.1	This study

## Declaration of Competing Interest

The authors declare that they have no known competing financial interests or personal relationships that could have appeared to influence the work reported in this paper.
